# *Mycobacterium leprae*-induced Insulin-like Growth Factor I attenuates antimicrobial mechanisms, promoting bacterial survival in macrophages

**DOI:** 10.1038/srep27632

**Published:** 2016-06-10

**Authors:** L. R. Batista-Silva, Luciana Silva Rodrigues, Aislan de Carvalho Vivarini, Fabrício da Mota Ramalho Costa, Katherine Antunes de Mattos, Maria Renata Sales Nogueira Costa, Patricia Sammarco Rosa, T. G. Toledo-Pinto, André Alves Dias, Danielle Fonseca Moura, Euzenir Nunes Sarno, Ulisses Gazos Lopes, Maria Cristina Vidal Pessolani

**Affiliations:** 1 Laboratory of Cellular Microbiology, Oswaldo Cruz Institute, Rio de Janeiro, 21040-900, RJ, Brazil; 2 Laboratory of Molecular Parasitology, Carlos Chagas Filho Institute, Federal University of Rio de Janeiro (UFRJ), Rio de Janeiro, 21940-590, RJ, Brazil; 3 Lauro de Souza Lima Institute, Bauru, 17034-971, SP, Brazil; 4 Leprosy Laboratory, Oswaldo Cruz Institute, Rio de Janeiro, 21040-900, RJ, Brazil

## Abstract

*Mycobacterium leprae* (ML), the etiologic agent of leprosy, can subvert macrophage antimicrobial activity by mechanisms that remain only partially understood. In the present study, the participation of hormone insulin-like growth factor I (IGF-I) in this phenomenum was investigated. Macrophages from the dermal lesions of the disseminated multibacillary lepromatous form (LL) of leprosy expressed higher levels of IGF-I than those from the self-limited paucibacillary tuberculoid form (BT). Higher levels of IGF-I secretion by ML-infected macrophages were confirmed in *ex vivo* and *in vitro* studies. Of note, the dampening of IGF-I signaling reverted the capacity of ML-infected human and murine macrophages to produce antimicrobial molecules and promoted bacterial killing. Moreover, IGF-I was shown to inhibit the JAK/STAT1-dependent signaling pathways triggered by both mycobacteria and IFN-γ most probably through its capacity to induce the suppressor of cytokine signaling-3 (SOCS3). Finally, these *in vitro* findings were corroborated by *in vivo* observations in which higher SOCS3 expression and lower phosphorylation of STAT1 levels were found in LL versus BT dermal lesions. Altogether, our data strongly suggest that IGF-I contributes to the maintenance of a functional program in infected macrophages that suits ML persistence in the host, reinforcing a key role for IGF-I in leprosy pathogenesis.

Leprosy manifests as a range of clinical forms in correlation with the type of immunological response generated during *Mycobacterium leprae* (ML) infection. As the result of a strong cellular immune response against ML, individuals with tuberculoid leprosy [polar tuberculoid (TT) and borderline tuberculoid leprosy (BT)] have few lesions and manifest a contained, self-limited infection in which scarce bacilli are detected. In contrast, patients with lepromatous leprosy [polar lepromatous (LL) and borderline lepromatous (BL)] display a progressively disseminated disease characterized by extensive bacterial multiplication within host cells and low cell-mediated immunity to the pathogen^1^. Although major advances have been made over the past several years, the mechanisms responsible for the permissive infection observed in LL individuals remain unclear.

ML is an intracellular pathogen preferentially found inside skin macrophages and Schwann cells in the peripheral nerves^1^. The cytokine patterns in tuberculoid and lepromatous lesions are distinct. While interferon-gamma (IFN-γ), tumor necrosis factor (TNF), and interleukin-15 (IL-15) predominate in tuberculoid skin lesions, lepromatous lesions are characterized by the expression of IL-10 and IL-4^2^,^3^. In the tuberculoid lesions, this cytokine profile favors the differentiation of classically-activated macrophages (epithelioid phenotype) accompanied by a high killing capacity and rare bacteria. Conversely, in the lepromatous lesions, macrophages are characterized by abundant intracellular bacilli due to their inefficiency in eliminating intracellular bacteria^4^. A significant portion of these macrophages shows a foamy phenotype as the result of lipid accumulation, referred to as Lepra or Virchow cells - a hallmark of LL lesions^5^. Complementing these data, a recent study correlated Virchow cell and tuberculoid lesion macrophage phenotypes with, respectively, the *in vitro* IL-10-induced macrophage phagocytic differentiation program and the IL-15-induced antimicrobial response^6^.

ML grows abundantly in the athymic nu/nu mouse footpad, producing granulomas resembling those found in human LL^7^. Previous studies have shown that, isolated from these granulomas, ML-burdened macrophages are defective in responding to activating signals, including IFN-γ, in addition to being incapable of killing intracellular pathogens^8^. *In vitro* ML-infected macrophages displayed a similar phenotype, indicating that the bacillus has the capacity to reprogram macrophage differentiation, which, in turn, contributes to the persistence of the bacillus in the host^9^. This effect has been shown to be at least partially mediated by the ML-induction of inhibitory levels of prostaglandin E_2_ (PGE_2_)^8^,^10^. However, to date, the molecular mechanisms involved in the ML subversion of macrophage microbicidal functions have only been partially deciphered.

Insulin-like growth factor I (IGF-I), a hormone with classic anti-apoptotic and proliferative activities, plays an essential role in tissue homeostasis^11^. Interestingly, a series of previous studies have pointed to IGF-I having a key role in experimental cutaneous leishmaniasis. It was shown that IGF-I induces an exacerbation of the lesion, thus favoring parasitic growth within host macrophages^12^. The ability of IGF-I to promote intracellular parasitic survival and proliferation was confirmed in *in vitro*-infected murine peritoneal macrophages. Upon IGF-I treatment of infected cells, it was demonstrated that this effect was linked to a concomitant increase in arginase activity together with decreased NO production and inducible nitric oxide synthase (iNOS) expression^13^.

Our group recently reported that ML induced the expression of IGF-I in human Schwann cells, thereby promoting host cell survival and delineating an important strategy for the successful colonization of ML in the nerve^14^. In the present study, we investigated the production of IGF-I by *in vitro* and *in vivo* ML-infected macrophages and its involvement in modulating macrophage microbicidal functions. Our results strongly suggest that the endogenous IGF-I produced by ML-infected macrophages inhibits the host cell antimicrobial mechanisms by blocking JAK/STAT-1-dependent signaling pathways activated in response to mycobacterial infection and IFN-γ stimulation.

## Results and Discussion

### The levels of IGF-I expression in dermal lesions of leprosy patients correlate with the outcome of the infection

Resident tissue macrophages demonstrate remarkable plasticity, which allows them to differentiate into a spectrum of functional phenotypes triggered by a complex mixture of signals originating from both the innate and adaptive immune responses. Based on their respective functions, these macrophages have been tentatively classified into M1 macrophages, involved in *host defense*, and M2 macrophages, a generic name for the various forms of macrophage activation devoted to *wound healing* and *immune regulation*^15^.

In leprosy, skin macrophages are major ML cell targets. In the case of LL lesions, instead of differentiating into classically-activated macrophages that combate and control the infection, skin macrophages actually allow for high bacillary replication levels, which then become excellent niches for bacterial persistence in the host. These extensively-studied macrophages have been shown to share characteristics with the regulatory anti-inflammatory phenotype. ML-infected macrophages become resistant to IFN-γ activation^8^, express higher levels of such scavenger receptors as CD36, SRA, and CD163^16^, and present a phenotype similar to the one induced *in vitro* by IL-10, which increases the phagocytic capacity of macrophages and leads to a reduction in bactericidal activity^6^.

**Figure 1:**
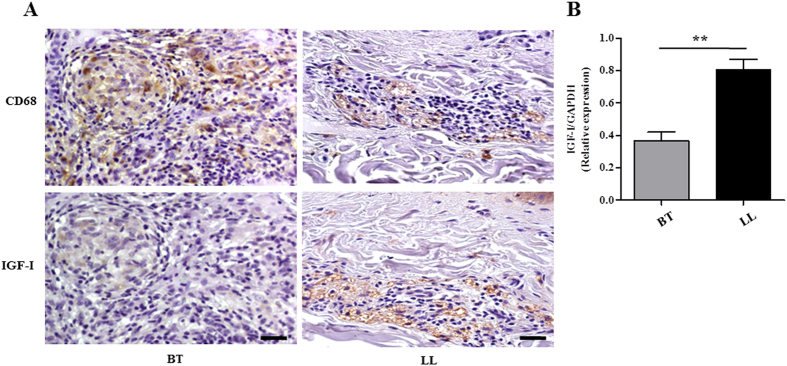
IGF-I is highly expressed in dermal lesions of lepromatous patients. (**A**) IGF-I and CD68 expressions were evaluated in serial sections of borderline (BT) and lepromatous (LL) skin lesions by immunohistochemical staining with diaminobenzidine. Immunolabelled cells appear as brown stained. Hematoxylin counterstained nuclei appear in blue. Data shown are representative of three lesions of each clinical form evaluated. Scale bar = 20 μm. (**B**) Comparative analysis of IGF-I mRNA expression in skin biopsies of BT (n = 7) and LL (n = 9) lesions by qRT-PCR. Data are shown as mean ± SE. Student’s t-test was performed and used for statistical analysis. **p < 0.005.

We started this study by comparing IGF-I expression levels in the skin lesions of multibacillary versus paucibacillary leprosy patients. Our hypothesis was that IGF-I levels would be higher in lesions with high bacterial numbers in contrast to lesions in which bacilli are rare. IGF-I levels were compared via immunostaining serial tissue sections of skin biopsy specimens taken from LL and BT patients with anti-IGF-I and anti-CD68, a macrophage marker. The immunoreactive cells appear as brown stained. Interestingly, LL skin lesions with anti-IGF-I exhibited much stronger labeling than the BT ones (Fig. 1A). Most IGF-I-positive cells presented a foamy appearance and CD68 positivity, representing macrophages with a Virchow cell phenotype. In BT samples, even though the epithelioid cells occupying the core of the typical tuberculoid granuloma were CD68 positive, they were found to stain negatively for IGF-I (Fig. 1A). The higher IGF-I expression in LL skin lesions was confirmed by qRT-PCR. The expression levels of the IGF-I mRNA were 2.2-fold higher (*p* = 0.0019) in LL than BT skin lesions (Fig. 1B). These data imply that ML infection induces the expression of IGF-I in *in vivo*-infected macrophages, which is in agreement with the outcome of the infection.

### IGF-I inhibits mycobacterium-induced iNOS expression and NO production in murine macrophages, promoting bacterial survival

Nitrogen reactive intermediates (NRI) have been shown to play an important role as effective microbicidal agents against mycobacteria in the experimental murine model^17^,^18^,^19^. Since IGF-I was previously shown to inhibit the generation of NRI in favor of parasitic growth in Leishmania-infected murine macrophages^12^, we first investigated whether a similar IGF-I effect would be seen in murine mycobacterium-infected macrophages. For that purpose, we adopted the RAW 264.7 murine macrophage cell line, both a good NO producer and easily transfected cell line that has been extensively used in studying macrophage-intracellular pathogen interactions. As a first step, we tested whether ML was able to induce IGF-I in this *in vitro* model. To this end, cells were infected with live ML or treated with irradiated ML for 48 h; and IGF-I protein levels were then measured in culture supernatants by using a specific sandwich ELISA. Both live and dead ML were able to significantly increase IGF-I levels in macrophage supernatants (Fig. S1A), exhibiting better induction at a bacteria:cell ratio of 50:1. Based on these results, unless otherwise mentioned, all subsequent experiments were conducted with dead bacteria due to difficulties in obtaining freshly-isolated ML from infected tissues. Interestingly, *M. bovis* BCG (BCG), another slow-growing attenuated mycobacterium that can grow inside macrophages^20^, was also able to induce IGF-I secretion in RAW cells. This was not, however, observed in macrophages treated with the fast-growing avirulent *M. smegmatis* (MS), which does poorly in these cells (Fig. S1B)^21^.

**Figure 2:**
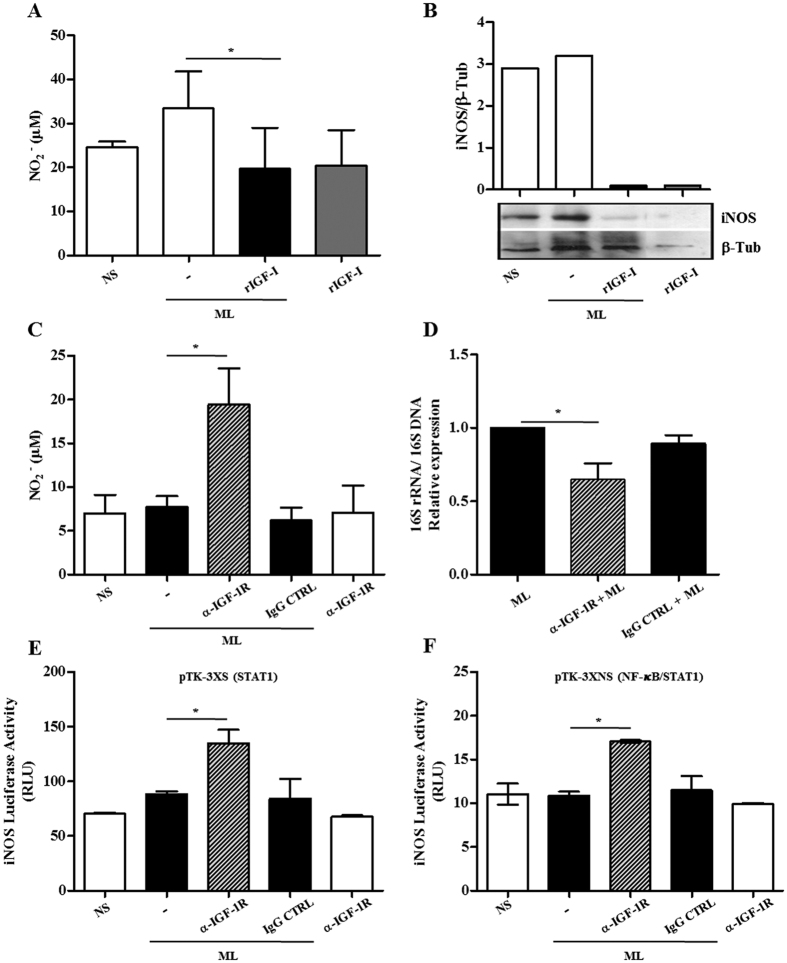
Blocking IGF-I signaling rescues antimicrobial activity in *M. leprae* (ML)-infected macrophages. (**A**) RAW 264.7 cultures were pre-treated with rIGF-I (50 ng/ml) for 30 min and then exposed to irradiated ML at a bacteria: cell ratio (50:1). After 48 h stimulation, nitrite in the supernatants was measured by the Griess reagent. Each value represents the mean ± SE of three independent experiments performed in duplicate. (**B**) Total lysates from cultures were subjected to Western blot using the specific antibodies against iNOS and β-tubulin (β-Tub). The Figure shows a representative Western blot from three independent experiments. (**C**) Nitrite amount in supernatants from murine macrophages pre-treated with a neutralizing antibody against IGF-1R (α-IGF-1R) for 30 min and then exposed to irradiated ML (50:1) for 48 h. Data are shown as mean ± SE from three independent experiments performed in duplicate. (**D**) ML viability measured by qRT-PCR using the ratio of 16S rRNA/16S DNA at 48 h in ML-infected macrophages pre-treated with α-IGF-1R. Data are shown as mean ± SE of three different experiments performed in triplicate. (**E,F**) RAW 264.7 cells transiently transfected with iNOS-luciferase reporter constructs pTK-3XS (**E**) or pTK-3XNS (**F**) were pre-treated with α-IGF-1R for 30 min and then exposed to irradiated ML (50:1) for 24 h. The cells were harvested and the luciferase activity determined by using the luciferase reporter assay system. Data are shown as mean ± SD of a representative experiment from three different ones performed in triplicate. An ANOVA test followed by Bonferroni as a post test were performed and used for statistical analysis. *p < 0.05.

We next analyzed the effects of IGF-I on NO release by these cells, as measured by the nitrite levels in culture supernatants. ML was unable to induce significant NO levels in the RAW 264.7 macrophage model (Fig. 2A), which was expected since it has been reported that ML is a poor activator of monocytes/macrophages^22^. Nonetheless, the addition of recombinant IGF-I (rIGF-I; 50 ng/ml) was able to dampen production of NO by about 25% in ML-treated cells (*p* < 0.05) (Fig. 2A). We also analyzed iNOS expression under the same conditions. And a reduction of the enzyme expression was observed in the presence of IGF-I (Fig. 2B).

Since the addition of exogenous rIGF-I had a relatively small impact on NO production under our assay conditions, and since ML was able to induce IGF-I in these cells, we then tested whether the inhibition of IGF-I signaling would bring about a greater increase in macrophage NO production in response to ML. IGF-I signaling was blocked with neutralizing antibody against the type 1 IGF-I receptor (α-IGF-1R) in ML-stimulated macrophages; and nitrite was measured in the culture supernatants. As displayed in Fig. 2C, when RAW 264.7 cells were incubated with α-IGF-1R, NO levels were about 2.5 times higher in response to ML (*p* = 0.0057). This was not observed when normal rabbit IgG was used in place of α-IGF-1R as a control. Moreover, as foreseen, a decrease of over 35% in ML viability was detected when RAW 264.7 cells were pretreated with α-IGF-1R in comparison to the cells treated with either control IgG or ML-infected cultures in the absence of any pretreatment (Fig. 2D) (p = 0.0155 and *p* = 0.0159, respectively).

The capacity of IGF-I to block the generation of NO was further confirmed by stimulating RAW 264.7 cells with BCG and MS, known as effective inducers of NO production^21^,^22^. Murine macrophages were pretreated with rIGF-I (50 ng/ml) and then stimulated with BCG or MS at a bacteria:cell ratio of 50:1. As expected, BCG and MS induced significant NO production when compared to unstimulated cells. However, pretreatment with rIGF-I reduced NO production to basal levels (Fig. S2A). The expression of the iNOS enzyme was also analyzed. Pretreatment with rIGF-I clearly inhibited BCG-induced iNOS expression in RAW 264.7 macrophages, paralleling NO production behavior (Fig. S2B). Interestingly, this type of reduction was not observed in rIGF-I pretreated, MS-stimulated macrophages. Finally, we investigated the effect of IGF-I on intracellular MS survival. As shown in Supplementary Figure 2C, cell pretreatment with rIGF-I was able to promote about 2.4 increase in MS intracellular survival in RAW 264.7 macrophages after a 48 h infection (p < 0.05). The initial growth of MS inside RAW cells during the first 24 h of infection is in agreement with the literature^21^. Combined, these results strongly indicate that IGF-I is able to down-modulate the NO/iNOS pathway in murine mycobacterial-infected macrophages, a major antimicrobial pathway, favoring intracellular bacterial survival.

### IGF-I blocks iNOS promoter expression regulated by STAT-1

The signal transducer and activator of transcription 1 (STAT1) and nuclear factor kappa B (NF-κB) are transcriptional factors that play a major role in controlling *iNOS* gene expression regulated by cytokines and pattern recognition receptors (PRR) ligands^23^. To investigate the capacity of IGF-I to interfere in any of these pathways, RAW 264.7 cells were transfected with iNOS promoter-luciferase reporter constructs containing responsive elements only for STAT1 (the pTK-3XS construct) OR for STAT1 and NF-kB (the pTK-3XNS construct). Expectedly, no effect on luciferase expression was observed in the transfected cells treated with ML alone. However, by blocking IGF-I signaling in the cultures, ML was able to significantly restore iNOS promoter activity in both the pTK-3XS (∼85% over baseline levels, *p* = 0.0099) and the pTK-3XNS construct (∼58% over baseline levels, *p* = 0.0024), as shown in Fig. 2E,F, respectively.

Similar experiments were conducted with BCG and MS in the luciferase reporter transfected cells. As shown in Supplementary Figure 2D,E, luciferase expression was highly induced by BCG and MS. Interestingly, rIGF-I pretreatment completely blocked mycobacterium-induced luciferase expression in the pTK-3XS construct. This did not occur, however, when NF-κB responsive elements were included in the promoter sequence (the pTK-3XNS construct), strongly indicating a specific effect of this hormone on JAK/STAT-dependent signaling pathways. Altogether, these results suggest that IGF-I blocks STAT-1-dependent, but not NF-κB-dependent signaling pathways triggered by mycobacteria in macrophages.

The capacity of mycobacteria to induce NO production in murine macrophages through a JAK/STAT-dependent signaling pathway has been previously described^24^,^25^. However, unlike BCG and MS, known to activate the NF-κB transcriptional factor^22^,^26^, ML has been shown to be both a weaker stimulator and even an inhibitor of NF-κB activation^22^,^27^,^28^. This observation might explain the similar inhibitory effect of IGF-I in the context of ML stimulation on iNOS promoter activity under the control of STAT1, whether in the presence or not of NF-κB responsive elements (observed by the higher activity of the promoter after blocking IGF signaling).

### IGF-I inhibits IFN-γ signaling in murine macrophages

IFN-γ is a key cytokine involved in controlling infections by intracellular pathogens such as *M. tuberculosis* and ML through activation of macrophage antimicrobial mechanisms like the NO/iNOS pathway in mice^29^,^30^. Since IFN-γ signals via a JAK/STAT pathway, and IGF-I was able to block a JAK/STAT-dependent pathway triggered by mycobacteria (Fig. 2E,F, Fig. S2D), as a next step, it was decided to investigate whether IGF-I contributes to the attenuation of IFN-γ signaling in murine macrophages.

**Figure 3:**
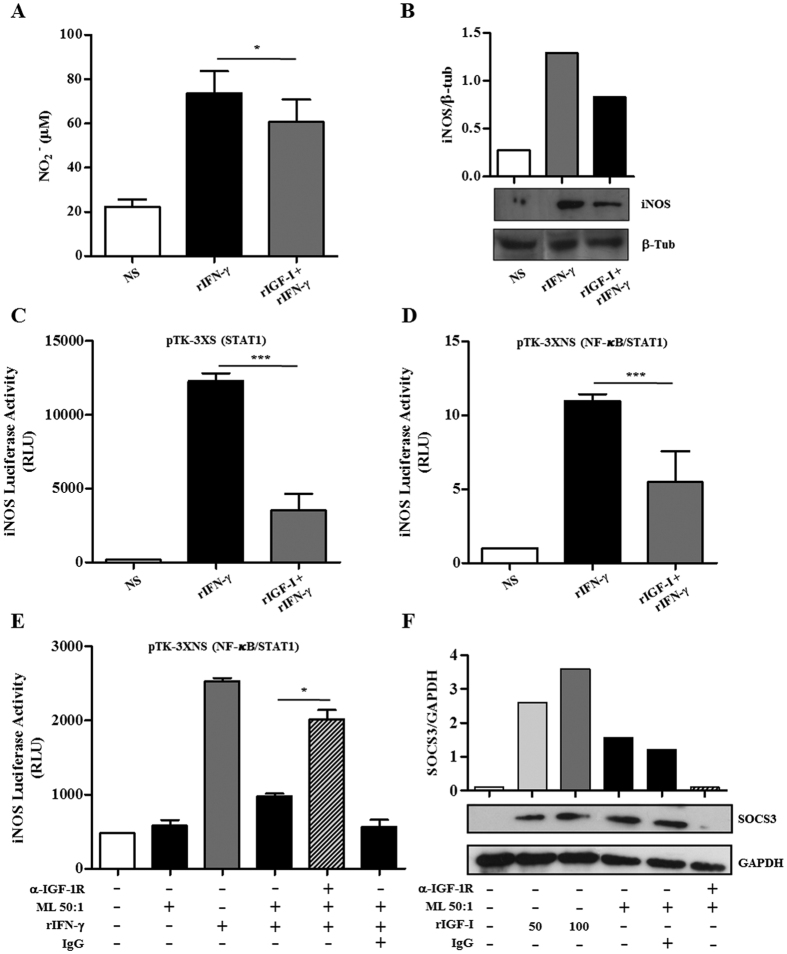
IGF-I inhibits IFN-γ signaling in murine macrophages. (**A**) RAW 264.7 cultures were pre-treated with rIGF-I for 30 min and then stimulated with IFN-γ (10 ng/ml). Nitrite amount in 48 h culture supernatants was measured by the Griess reagent. Data are shown as mean ± SE from three independent experiments performed in duplicate. (**B**) Western blot analysis of 24 h RAW 264.7 cultures using the specific antibodies against iNOS and β-tubulin (β-Tub). The figure shows a representative Western blot from three independent experiments. (**C,D**) RAW 264.7 cells transiently transfected with the iNOS-luciferase reporter constructs pTK-3XS (**C**) or pTK-3XNS (**D**) were pre-treated with rIGF-I for 30 min and then stimulated with rIFN-γ for 24 h. The cells were harvested and luciferase activity determined by using the luciferase reporter assay system. Data are shown as mean ± SD of a representative experiment from three different experiments performed in triplicate. (**E**) RAW 264.7 cells transiently transfected with the iNOS-luciferase reporter construct pTK-3XNS were pre-exposed to ML (50:1) or ML plus neutralizing antibody against IGF-1R (α-IGF-1R) for 1 h and then stimulated with IFN-γ for 24 h. Unstimulated cultures were included as a control. The cells were harvested and luciferase activity determined by using the luciferase reporter assay system. (**F**) RAW 264.7 macrophages were exposed to irradiated ML (50:1), or stimulated with rIGF-I at different concentrations (50 or 100 ng/ml), or ML plus neutralizing antibody α-IGF-1R for 24 h. Western blot analysis was performed using the specific antibodies against SOCS3. GAPDH was used as a loading control. The Figure shows a representative Western blot from three independent experiments. Data are shown as mean ± SD of a representative experiment from three different experiments performed in triplicate. An ANOVA test followed by Bonferroni as a post test were performed and used for statistical analysis. *p < 0.05, ***p < 0.0001. RLU, relative luminescence units.

We initially evaluated the capacity of IGF-I to inhibit IFN-γ signaling by measuring NO release and iNOS expression in RAW 264.7 macrophages pretreated or not with rIGF-I and stimulated with rIFN-γ. As shown in Fig. 3A, a reduction of about 28% (*p* < 0.0001) in rIFN-γ-induced NO production was observed in rIGF-I-treated cells. This result paralleled the capacity of rIGF-I to inhibit the rIFN-γ-triggered expression of iNOS (Fig. 3B). The inhibition of rIFN-γ-induced iNOS expression by rIGF-I was also observed in RAW 264.7 cells transfected with the iNOS promoter-luciferase reporter constructs pTK-3XS (about 68%, *p* < 0.0001) and pTK-3XNS (~54%, *p* *<* 0.0001), as shown in Fig. 3C,D, respectively. These data clearly show that IGF-I is able to downregulate IFN-γ-mediated STAT1 activation pathway in RAW macrophages. These results are in line with a recent report that showed that a deficit in IGF-I/Akt signalling in murine macrophages is associated with an increase in responsiveness to IFNγ^31^. Reiforcing the idea that IFN-γ and IGF-I may have antagonistic functions, it has been previously shown that IFN-γ inhibits IGF-I expression by macrophages^31^,^32^.

Next, the potential involvement of IGF-I in the well-known refractory phenotype of ML-infected macrophages to IFN-γ activation^8^,^10^ was analyzed. As shown in Fig. 3E, the capacity of ML-stimulated macrophages transfected with the iNOS-promoter reporter pTK-3XNS construct to respond to rIFN-γ was shown to be impaired [activity was reduced to about 2.5 (*p* = 0.0001)] when compared to cells not treated with ML. Of note, blocking IGF-I signaling via α-IGF-1R partially restored the capacity of rIFN-γ to activate the iNOS promoter in ML-stimulated macrophages (activity roughly doubled in comparison to that of cells treated with ML and rIFN-γ alone (*p* = 0.0014). These data suggest that IGF-I plays a key role in the deactivated phenotype of ML-infected macrophages. However, complementary assays measuring NO release and bacterial viability are necessary to confirm this hypothesis.

Lastly, the underlying mechanism responsible for the inhibitory effect of IGF-I on STAT1 signaling in the murine macrophage model was explored. SOCS1 and SOCS3 are classical inhibitors of the JAK/STAT signaling pathway and negatively regulate both the innate and adaptive immune responses^33^. Since the capacity of IGF-I to induce SOCS3 has been previously described^34^, the possible involvement of SOCS3 in the inhibitory effect of IGF-I over the STAT1 signaling pathway was investigated. For this purpose, the modulation of SOCS3 expression by ML and IGF-I in RAW macrophages was investigated. Interestingly, as shown in Fig. 3F, after 24 h, both ML and rIGF-I were able to increase SOCS3 expression in RAW 264.7 macrophages. Besides, the blockage of IGF-I signaling led to the elimination of SOCS3 induction by ML, indicating that ML induces SOCS3 via IGF-I (Fig. 3F). We can speculate that the induction of SOCS3 by IGF-I may be mediated by IL-10, a cytokine known to induce SOCS3^35^,^36^, since both ML and IGF-I promote IL-10 production by monocytes^16^,^22^. These results point to the potential role of SOCS3 in the IGF-I inhibitory effect on JAK/STAT signaling in ML-stimulated macrophages.

### Dampening IGF-I signaling increases ML intracellular killing in human macrophages

**Figure 4:**
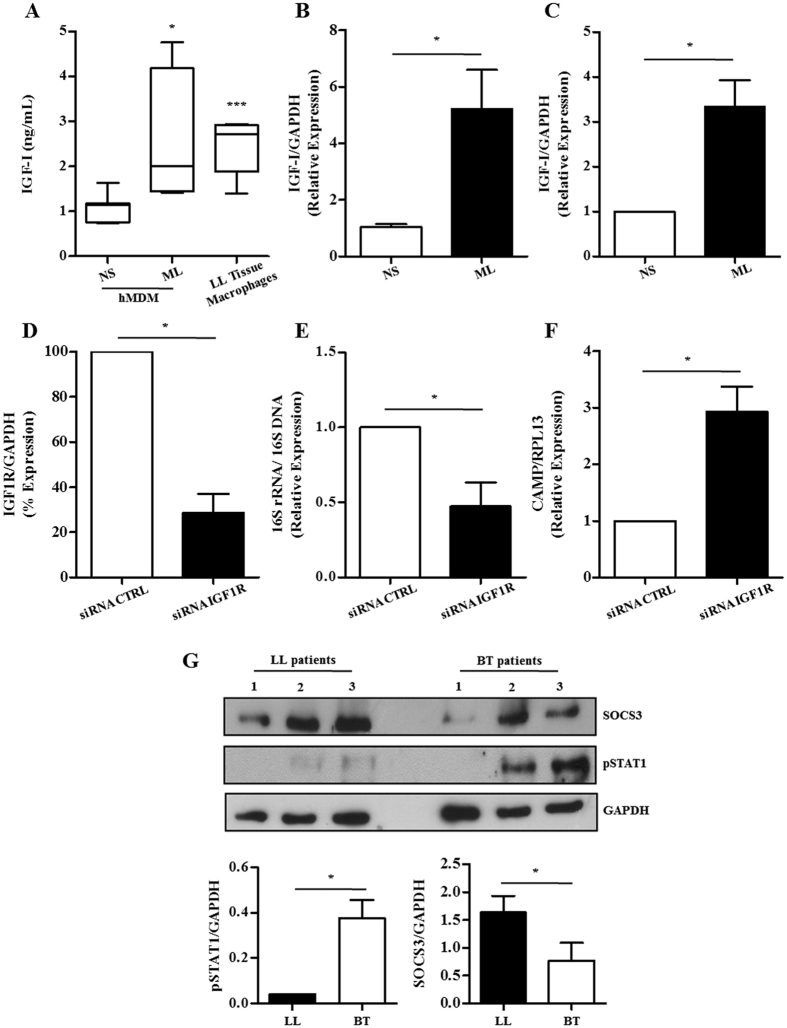
Knockdown of IGF-1R rescues antimicrobial activity in ML-infected human macrophages. (**A**) IGF-I protein levels assessed by specific sandwich ELISA in culture supernatants of *in vitro* ML-infected hMDM (n = 8) or *in vivo* ML-infected macrophages from LL skin lesions (LL Tissue Macrophages; n = 5). (**B–C**) IGF-I mRNA levels assessed by qRT-PCR in hMDM cultures (**B**) or THP-1 human macrophages (**C**) stimulated with irradiated ML (50:1) for 24 h. (**D**) THP-1 macrophages were transfected with siRNA IGF1R or non-targeting siRNA (siRNA CTRL) and 24 h later were infected with ML for additional 48 h. The effect of siRNA against IGF-1R was measured by qRT-PCR. (**E**) ML viability measured by qRT-PCR using the ratio of 16S rRNA/16S DNA in ML-infected IGF1R THP-1 knockdown macrophages. (**F**) Cathelicidin (*CAMP*) mRNA levels were assessed by qRT-PCR in IGF1R THP-1 knockdown macrophages infected with ML. (**G**) Western blot analysis of SOCS3 and pSTAT1 expression in LL (n = 3) and BT (n = 3) skin lesions of leprosy patients. GAPDH was used as a loading control. The bar graph represents the densitometric analysis of SOCS3 and pSTAT1 (mean values ± SE). Data are shown as mean ± SD of a representative experiment from three different experiments performed in triplicate. An ANOVA test followed by Bonferroni as a post test were performed and used for statistical analysis. *p < 0.05, ***p < 0.0001.

So far, we have shown that IGF-I induced during ML infection is able to interfere with the NO/iNOS pathway, a major antimicrobial mechanism in murine macrophages. Since the vitamin D-cathelicidin pathway, but not the NO/iNOS one, seems to represent a major mechanism used by human macrophages to kill mycobacteria^37^, next we tested the effect of this hormone on this pathway in human cells. Firstly, *ex vivo* and *in vitro* experiments were conducted to confirm the capacity of ML to induce IGF-I in human macrophages, as suggested by the immunostaining analysis of leprosy skin biopsies (Fig. 1A). Since the inflammatory infiltrate in LL lesions is enriched in infected macrophages, fresh biopsies were dissociated. Moreover, tissue macrophages were recovered in relatively good numbers and their spontaneous IGF-I secretion was monitored in culture supernatants after 48 h of incubation at 33 °C via ELISA. These cells are shown microscopically in Supplementary Figure 3. Culture visualization of these cells by DIC revealed collections of bacteria within cells with a foamy aspect resembling that of Virchow cells. In parallel, human monocyte-derived macrophages (hMDM) were obtained from healthy donors and infected with ML freshly isolated from LL skin lesions in an attempt to mimick the *in vivo* condition. As shown in Fig. 4A, the IGF-I levels detected in the conditioned media of *in vivo*-infected macrophages and *in vitro* ML-infected hMDM were similar (2.46 ± 0.7 ng/ml in tissue macrophage cultures versus 2.54 ± 0.2 ng/ml in ML-infected hMDM cultures) and yet significantly higher than the levels produced by unstimulated hMDM (1.06 ± 0.1 ng/ml; *p* *=* *0.0002 and p* *=* *0.021*, respectively). IGF-I regulation was shown to be at the transcriptional level since hMDM incubated with ML showed significantly higher levels of IGF-I transcripts (5.2-fold higher, *p* = 0.0009) in comparison to the control cells (Fig. 4B).

Next, to ascertain the role of IGF-I in intracellular ML survival in human macrophages, we used small interfering RNA (siRNA) technology to knockdown transcripts of the IGF-1R receptor (siRNA IGF1R). The human monocytic cell line THP-1 was selected because of its high transfection efficiency with siRNAs. Furthermore, THP-1 cells have been shown to activate the vitamin D-dependent antimicrobial response against mycobacteria^37^ and, to express higher levels of IGF-I transcripts in response to ML (by about 3.5 fold higher, *p* = 0.0108) (Fig. 4C), similarly to primary cells (Fig. 4A). Transfection of siRNA IGF1R into THP-1 cells resulted in about a roughly 70% decrease in IGF-1R mRNA levels (*p* = 0.013), as determined by qRT-PCR, when compared to cells transfected with a non-targeting siRNA (siRNA CTRL) (Fig. 4D), without any apparent effect on cell viability. To address the role of the IGF-I pathway in ML survival in human macrophages, THP-1 cells transfected with siRNA IGF1R or siRNA CTRL were infected with ML at a MOI of 50:1 for 48 h; and ML viability was assessed by qRT-PCR, as described above. About 50% decrease (*p* = 0.03) in ML viability was observed in IGF-1R siRNA transfected cells when compared to cells transfected with siRNA CTRL (Fig. 4E). Of note, the accelerated killing of ML observed in IGF-1R knockdown cells positively correlated with the expression of the antimicrobial peptide cathelicidin (2.9 fold higher, *p* = 0.012) (Fig. 4F). These data indicate that the IGF-I signaling pathway plays a role in bacterial intracellular survival in human macrophages by attenuating the vitamin D-cathelicidin innate immune pathway.

As a final step, in an effort to make our data more physiologically relevant, we prepared protein extracts of leprosy patient skin biopsies and investigated the relative levels of SOCS3 expression and the phosphorylated form of STAT1 (pSTAT1). Increased SOCS3 levels were found in the multibacillary lepromatous lesions as compared to the paucibacillary tuberculoid ones (Fig. 4G). In contrast, lower levels of pSTAT1, an indication of decreased JAK/STAT signaling pathway activity, were detected in LL lesions than BT ones (Fig.4G). These data are in agreement with the IGF-I expression levels in these lesions and reinforce the *in vitro* results obtained in the RAW model, suggesting that, during the natural infection in humans, this hormone dampens JAK/STAT1-dependent signaling pathways in macrophages via SOCS3 induction. However, new experiments are necessary to confirm the role of SOCS3 in leprosy pathogenesis. SOCS3 inhibits pro-inflammatory cytokine signals, including IFN-γ, through direct interaction with JAK1, JAK2, and TYK2^38^. Of note, it has been recently shown that a SOCS3 deficiency promotes M1 macrophage polarization, indicating that SOCS3 plays a key role in the modulation of classical macrophage activation^39^. Similarly, the expression of SOCS by *M. tuberculosis*-stimulated peripheral blood mononuclear cells (PBMC) has been linked to the clinical severity of tuberculosis^40^.

The central events in the regulation of the immune response, especially in chronic human diseases, occur within the tissues. Based on the data presented herein, we propose that IGF-I actively participates in the regulatory phenotype of ML-infected macrophages found in lepromatous lesions. ML-infected macrophages have been seen to be good producers of PGE_2_ and IL-10 both *in vivo* and in *vitro*^16^,^22^,^41^ and the production of these mediators has been associated with the unresponsiveness of these cells to IFN-γ signaling^9^. It has been shown that IGF-I induces IL-10 production in monocytes^42^, implicating IGF-I as a primary contributory signal to IL-10 secretion in ML-infected macrophages. IL-10 and PGE_2_, in addition to other factors, may provide a signal for the differentiation of regulatory macrophages^15^,^43^ in LL/BL lesions and would also inhibit Th1 response. Besides, IL-4, a potent IGF-I inducer^44^, also abundant in LL/BL lesions, would help maintain the high IGF-I levels in these lesions. In contrast, a different scenario is found in tuberculoid leprosy lesions, in which macrophages are in a Th1 environment with a predominant production of IFN-γ. IFN-γ promotes the classical activation of macrophages and favors the secretion of inflammatory mediators and antimicrobial effector molecules while inhibiting IGF-I production.

In a previous study, we found normal circulating levels of IGF-I in patients with different clinical forms of leprosy. The only exception was a group of LL/BL patients who had never developed a reactional episode during the course of the disease and whose IGF-I levels were significantly lower^45^. Although apparently contradictory to our current findings, the fact that circulating IGF-I was measured in the earlier study, in contrast to the skin lesion levels analyzed in the present report, could explain the differences observed. IGF-I is produced by most bodily tissues, acting in both autocrine and paracrine ways and affecting local cell physiology and metabolism. This production is regulated by several factors present in the tissue microenvironment. In contrast, over 80% of circulating IGF-I is produced by the liver and controlled by a complex immune neuroendocrine regulatory system^46^. We propose that the higher IGF-I expression observed in the skin lesions described herein is driven by the presence of ML and the local inflammatory response.

A question that remains unanswered, however, is precisely how ML induces IGF-I, which appears to be an early event during ML-macrophage interaction. Since non-viable bacteria is required, we propose that IGF-I induction is mediated by the recognition of preexisting pathogen-associated molecular pattern (PAMPs) by their corresponding host cell pattern recognition receptors (PRRs). Indeed, it was recently shown that CpG oligodeoxynucleotides, classical ligands of TLR9, are able to induce IGF-I in intestinal epithelial cells^42^. Leprosy is a chronic disease where, in lesions of untreated LL/BL patients, it is believed that a proportion of the bacilli is dead^47^. Therefore, pathways triggered by ML PAMPs, independently of bacterial viability, which could attenuate local inflammation and contribute to bacterial survival, might play an important role on infection persistence. The capacity of live ML to induce IGF-I would be most relevant at early stages of the infection, but the ability of dead bacteria to do the same would gain importance in the chronic phase of the disease.

Moreover, based on our data it is reasonable to propose that the levels of IGF-I produced by macrophages in response to ML early during infection could impact the course of disease progression, contributing to different clinical outcomes. In the present study, we limited the analysis to the effect of IGF-I on macrophage anti-bacterial mechanisms. However, a recent report showed that IGF-I promotes the induction of M2 macrophage markers^31^, suggesting that IGF-I may be a key element involved in the polarization of macrophages at the very early phase of ML infection affecting disease outcome. Leprosy has a strong genetic influence, and single nucleotide polymorfisms (SNPs) in several genes of the innate and adaptive immune responses have been associated with susceptibility to leprosy *per se* or to the severity of the disease^48^. So, it is reasonable to speculate that mutations that may affect the functional levels of IGF-I at the site of ML infection may influence the susceptibility to leprosy and disease progression to either the self-limited tuberculoid forms (low IGF-I producers) or to the disseminated infection found in lepromatous patients (high IGF-I producers). Future genetic studies on the association of mutations in genes involved in IGF-I expression or even in genes of the IGF-I system with leprosy susceptibility will clarify this point.

In conclusion, based on *in vitro* experiments complemented by leprosy skin lesion analyses, our data strongly suggest that IGF-I contributes to the permissive phenotype found in infected macrophages in LL lesions, favoring intracellular bacterial persistence and a chronic course for leprosy. The higher IGF-I levels observed in LL lesions may be critical in leprosy pathogenesis by facilitating bacterial persistence through inhibition of the antimicrobial mechanisms activated by the JAK/STAT signaling pathway. Our findings are in line with a recent report, in which the role of IGF-I in inducing an immunosupressive phenotype in monocytes in the context of a chronic inflammatory disease was demonstrated^42^. This study showed that mouse intestinal epithelial cell-derived IGF-I can induce IL-10-expressing monocytes in the intestine. Also, the IGF-primed monocytes were shown to inhibit inflammation in a model of experimental colitis in mice. Moreover, our data extend the capacity of IGF-I to promote growth/survival of another intracellular parasite within the host, initially demonstrated in the context of *Leishmania*^12^,^49^. In addition, it has been demonstrated that the *in vivo* administration of IGF-I signaling inhibitors had a therapeutic effect in a murine model of *L. donovani* infection when combined with conventional anti-leishmanial chemotherapy^50^. Again, based on our findings, a similar rationale could be applied to leprosy, potentially opening new avenues for novel therapeutic strategies to treat the disease.

## Methods

### Patients and clinical specimens

Thirty-three leprosy patients with no signs of a reactional episode (13 BT and 20 LL consisting of 15 men and 18 women between the ages of 25–45) were classified according to the Ridley and Jopling scale^51^ and included in this study. Skin biopsy specimens (6 mm punch) were obtained before treatment and used for immunohistochemical, Western blot and transcriptional analyses. Buffy coats were obtained from healthy donors at the Hemotherapy Service of the Clementino Fraga Filho Universitary Hospital, associated with the Federal University of Rio de Janeiro, RJ, Brazil. The present study was conducted in accordance with the World Medical Association’s Declaration of Helsinki. All protocols and the acquisition of all specimens were approved by the Ethics Committee of the Oswaldo Cruz Foundation (FIOCRUZ, Rio de Janeiro, RJ, Brazil) and the Lauro de Souza Lima Institute (Bauru, São Paulo, SP, Brazil). Signed informed consent was voluntarily obtained from each participant.

### Mycobacteria

ML (viable and lethally-irradiated) derived from the footpads of athymic *nu/nu* mice was kindly provided by Patrícia Samarco Rosa (Lauro Souza Lima Institute). *M. bovis* BCG Pasteur (ATCC35734) and *M. smegmatis* (mc^2^ 155) strains were grown at 37 °C in Middlebrook 7H9 broth medium (Becton Dickinson, Sparks, MD, USA) supplemented with 10% OADC (v/v) and 0.05% Tween 80 (v/v) under constant agitation on a magnetic plate until the exponential phase, counted according to Shepard and McRae^52^, and kept frozen at −70 °C until use. Addicionally, ML was isolated from the skin nodules of LL patients as detailed below.

### Cell cultures and stimulation

The murine macrophage cell line RAW 264.7 and the human monocytic THP-1 was purchased from American Type Culture Collection (ATCC), maintained in RPMI 1640 medium containing 2 mM L-Glutamine (LGC Biotecnologia, Cotia, SP, Brazil), and supplemented with 10% FCS (CULTILAB, Campinas, SP, Brazil) and the antibiotics penicilin and streptomycin (LGC Biotecnologia). Cultures were kept at 37 °C within a humidified 5% CO_2_ atmosphere.

Cells were plated in complete RPMI medium at a density of 40 000 or 10^6^ cells in 24- or 6-well plates, respectively, and allowed to attach for 6 h at 37 °C with 5% CO_2_. THP-1 cell differentiation into macrophages was induced by 80 nM of PMA incubated at 37 °C for 24 h. Recombinant IGF-I (50 ng/ml; R&D Systems, Minneapolis, MN, USA) was added 30 min before the mycobacterial species or recombinant IFN-γ (10 ng/mL; R&D Systems, Minneapolis, MN, USA) stimuli. The dose of IGF-I used in our assays was based on a previous study (Rodrigues *et al*.^14^). Unstimulated cultures were included as control**s**.

For primary monocytic isolation, peripheral blood mononuclear cells (PBMCs) from healthy volunteers were isolated under endotoxin-free conditions from buffy coats by density sedimentation over Histopaque-1077 (Sigma, St Louis, MO, USA). PBMCs were allowed to adhere to culture flasks (Nunc, NY, Rochester, USA) for 1 h in serum-free RPMI medium at 37 °C; and the non-adherent cells were removed by vigorous washing with PBS. The recovered cells were composed of 95% monocytes, as determined by flow cytometric analysis. Human monocyte-derived macrophages (hMDM) were prepared by culturing peripheral blood monocytes plated in complete RPMI medium at 37 °C for 7 days with 5% CO_2_.

### Isolation of ML and macrophage cells from skin biopsies

Five skin biopsies (6-mm diameter) were obtained from the LL patients before treatment and processed, as previously described by Moura et al.^53^, at Souza Araujo Outpatient, FIOCRUZ, Rio de Janeiro, RJ, Brazil. The specimens were placed in a tube with RPMI 1640 medium on ice. The epidermis was gently withdrawn with a razor blade and the dermis minced and incubated overnight at 33 °C in a humid atmosphere with 5% CO_2_ in an enzyme mixture (0.5 mg/mL of collagenase and 4 mg/mL of dispase; (Invitrogen Corporation, Carlsbad, CA, USA) in RPMI medium with 10% FBS (Hyclone, Logan UT, USA). The digested tissue was centrifugated at 1,500× g for 10 min and the supernatant was saved for ML isolation. The pellet containing the macrophages was ressuspended in RPMI with 10% FBS; and 5 × 10^5^ cells were added to 24-well plates and incubated at 33 °C in a humid atmosphere with 5% CO_2_ for up to 2 days. Purity of the macrophage cultures was determined by flow cytometry (BD Accuri C6, BD Biosciences). ML was recovered from the tissue supernatant by centrifugation at 10,000× g for 15 min. Bacteria were treated with 0.1 N NaOH for 3 min at room temperature to remove tissue contaminants and washed twice with RPMI medium. The final pellet was ressuspended in RPMI medium; and bacterial clamps were dissociated and counted according to Shepard and McRae^52^.

### Transfection and gene silencing experiments

Gene expression knockdown was achieved in THP-1 cells by transfection with pre-designed Silencer Select siRNA IGF-1R (code 48494812/13-IDT) or siRNA negative control (code 4390874 – Ambion), Lipofectamine 2000 (Life Technologies) was used as vehicle according with manufector’s instructions. The cells were plated in a 24-well plate and transfected with 20 pmol of siRNA in a 100ul transfection mix of Opti-MEM (Gibco) and lipofectamine. The protocol is according to the manufacturer’s instructions (Life technologies). Twenty-four hours following the transfection, cells were infected with live *M. leprae* for 48 h, after which nucleic acids were extracted and mRNA expression was measured by qRT-PCR.

### Immunohistochemical studies

Skin biopsies of LL and BT patients were obtained at diagnosis and prior to treatment at the Lauro de Souza Lima Institute, Bauru, São Paulo, SP, Brazil. For routine histopathological analyses, all skin tissues were stained with hematoxylin and eosin (H&E) to estimate the extent and composition of the inflammatory infiltrate. Wade stains were utilized to evaluate the bacillary load^54^. Immunostaining was performed in serial sections on the same slides of each specimen by incubation with rabbit anti-IGF-I (GroPep, Thebarton, Australia) (1:100) or mouse anti-CD68 (DAKO, Denmark) (1:50) in 10 mMTris/HCl buffer, pH 7.4, containing 1% bovine serum albumin. Negative controls were performed by using normal rabbit mouse IgG and suppression of primary antibodies. After overnight incubation in a humidified chamber at 4 °C, sections were rinsed with TBS/0.1% Triton X-100 and incubated in secondary antibodies with anti-rabbit or anti-mouse specificity and then conjugated to a dextran polymer backbone in which multiple horseradish peroxidase molecules were attached (EnVision^+^ System, DAKO). Immunoreactions were stained with 3,3′-diaminobenzidine (Liquid DAB^+^ Chromogen, DAKO) and, after immersion in distilled water, sections were counterstained with Mayer’s hematoxylin, followed by dehydration and covering by a permanent mounting medium (DAKO). Images were obtained via a Nikon Eclipse microscope with Image ProPlus software.

### Cellular immunofluorescence

The Virchow cells isolated from LL skin lesions were fixed in PFA 4% and then incubated with the anti-LAM primary antibody [kindly provided by Dr. Patrick J. Brennan (Colorado State University, Fort Collins, CO, USA; National Institutes of Health/National Institute of Allergy and Infectious Diseases, Contract No. 1Al25469] for 2 h at room temperature. Cells were washed extensively with PBS and then incubated with IgG goat anti-mouse secondary antibody conjugated to Alexa-488 (Molecular Probes; 1:250) for 1 h at room temperature. The images were obtained from the Axio Observer Z1 Microscope (Carl Zeiss, Göttingen, Germany) via Axiovision 4.7 software. Cytochemical staining was analyzed by differential interference contrast (DIC) and fluorescence microscopy. A mouse IgG1 (BD Biosciences) was used as a control.

### Real-time quantitative reverse transcription PCR (qRT-PCR)

Total RNA was extracted from frozen skin fragments or ML-stimulated cells using the Trizol reagent (Invitrogen), according to the manufacturer’s instructions. Total RNA was converted to cDNA using Oligo(dT) and Superscript III reverse transcriptase (Invitrogen), as instructed. Five micrograms of total RNA were reverse transcribed into cDNA and samples were stored at −20 °C until further use. qRT-PCR was performed using an ABI Prism 7000 Sequence Detection System (Applied Biosystems, Foster City, CA, USA). TaqMan Universal PCR Master Mix, specific primers [human IGF-I (Hs01555481_m10); human IGF-1R (Hs00951562_m1) and human cAMP (cathelicidin - Forward: GCTGGTGAAGCGGTGTATG; Reverse: TGCCAATCTTCTCTTTAGATTTCC)] were purchased from Applied Biosystems and used according to the manufacturer’s instructions. Thermal cycling conditions comprised an initial incubation at 50 °C for 2 min, 95 °C for 10 min, 40 cycles of denaturation at 95 °C for 15 s, and annealing and extension at 60 °C for 1 min. To normalize the relative expression of the genes of interest, Glyceraldehyde-3-phosphate dehydrogenase [human GAPDH (4333764-F)] was used as an endogenous control whereas the expression values obtained were corrected and quantified by converting the cycle threshold (Ct) value into a numerical one according to the following formula: expression value = 2^(−ΔΔct)^.

### Western blot analysis

Equal amounts of protein/cell lysate obtained from mycobacterial-stimulated cultures or slices (10 μm) of frozen patient skin biopsies (BT, n = 3 and LL, n = 3) were loaded onto a sodium dodecyl sulfate-polyacrylamide gel electrophoresis (SDS-PAGE) and transferred to nitrocellulose membranes (Bio-Rad, Hercules, CA, USA). Immunoblottings were developed with rabbit polyclonal anti-iNOS antibody (Santa Cruz), rabbit polyclonal anti-SOCS3 (Santa Cruz), rabbit polyclonal anti-pSTAT1 (Cell signaling Technology, Danvers, MA, USA), mouse monoclonal anti-STAT1 (Cell Signaling Technology), and anti-β-tubulin (Santa Cruz) in 5% (w/v) nonfat milk dissolved in Tris-Tween-buffered saline (TTBS; 20 mMTris-HCl buffer, pH 7.6, containing 137 mM NaCl–0.05% [v/v] Tween 20). Results were visualized via an enhanced chemiluminescence detection system (ECL; Amersham Biosciences, Psicataway, NJ, USA).

### Nitric oxide

Nitrite anion (NO_2_^−^) accumulation, used as an indicator of NO production, was determined in supernatants from mycobacterial-stimulated cultures by using Griess reagent. Briefly, equal volumes of culture supernatant and Griess reagent (50 μL) were mixed for 15 min at room temperature. Absorbance at 570 nm was measured via a Lab Systems Multiscan plate reader. A solution of sodium nitrite was used to construct a standard curve.

### Luciferase assay

As previously described, to investigate the activity of the iNOS promoter, RAW 264.7 macrophages (10^5^ cells per well) were plated in 48-well polystyrene plates^55^ and transfected via Lipofectamine 2000 reagent (Invitrogen) by way of the manufacturer’s instructions. Cells were transfected with 1 μg pTK-3XNS or pTK-3XS (kindly provided by Dr. David Geller) plus plasmid pRL-CMV (Promega) that was the co-reporter for the normalization of experimental variations. Cells transfected with pTK-3XNS or pTK-3XS and pRL-CMV were treated with IGF-I (50 ng/ml; R&D Systems), IFN-γ (10 ng/ml; R&D Systems), or anti-IGF-1R (5 μg/ml; R&D Systems). Unstimulated cultures were included as controls. After these treatments, cells were washed with PBS, lysed according to the Dual Luciferase System Protocol (Promega), and analyzed in the TD-20/20 Luminometer (Turner Designs, Sunnyvale, CA, USA). Luciferase activity was measured using the Luciferase Assay System (Promega), according to the manufacturer’s protocol. Each transfection was performed in triplicate, and three independent experiments were conducted.

### Determination of IGF-I protein levels

The levels of IGF-I in culture supernatants were determined by ELISA using the IGF-I Duo Set kit (R&D Systems) in accordance with the manufacturer’s instructions.

### Mycobacterial viability

Before infection, non-adherent RAW 264.7 macrophages were removed by washing with phosphate-buffered saline (PBS) (pH 7.2); and the medium was replaced by an antibiotic-free medium supplemented with 10% FBS. Cultures were treated or not with recombinant IGF-I (50 ng/ml) for 30 min, infected with viable ML or MS, and immediately centrifuged at 400× *g* for 10 min, followed by incubation at variable time points at 33 °C (ML) or 37 °C (MS). MS viability was assessed by the colony-forming unit (CFU). Briefly, infected macrophages were treated with gentamicin (200μg/mL) for 2 h to kill extracellular bacteria and then lysed with 0.1% Triton X-100. Cell lysates were subsequently serially diluted and plated onto a 7H10 medium supplemented with OADC. Colonies were counted after incubation at 37 °C for 3 days (MS)^56^. ML viability was determined by qRT-PCR, using the protocol previously described by Martinez, *et al*.^57^. Briefly, ML RNA and DNA were simultaneously extracted through a single-tube homogenization protocol using the Fast Prep FP 24 instrument (MP Biomedicals). DNA was removed from the RNA preparations using the DNA-free turbo kit (Ambion). RNA was reverse transcribed via random primer and superscript III following the manufacturer’s instructions (Invitrogen). The levels of 16S rRNA were determined and normalized against 16S DNA; and both were measured by a TaqMan real-time PCR assay. PCR conditions were the same as those described in the quantitative qRT-PCR section (above); and reactions were incubated in an ABI StepOne Plus Sequence Detection System (Applied Biosystems).

### Statistical Analysis

The results are expressed as mean ± SE and analyzed via the Student’s t-test when the means of two groups were compared. ANOVA was applied for multiple comparisons followed by the Bonferroni as a post-hoc test. Differences were considered significant at p < 0.05. Data analysis was performed using the GraphPad Prism 5.0 (GraphPad Software, Inc., San Diego, CA, USA).

## Additional Information

**How to cite this article**: Batista-Silva, L. R. *et al*. *Mycobacterium leprae*-induced Insulin-like Growth Factor I attenuates antimicrobial mechanisms, promoting bacterial survival in macrophages. *Sci. Rep.* **6**, 27632; doi: 10.1038/srep27632 (2016).

## Supplementary Material

Supplementary Information

## References

[b1] Scollard D. M. . The continuing challenges of leprosy. Clin. Microbiol. Rev. 19, 338–381 (2006). 16614253 10.1128/CMR.19.2.338-381.2006 PMC1471987

[b2] Yamamura M. . Defining protective responses to pathogens: cytokine profiles in leprosy lesions. Science 254, 277–279 (1991). 1925582 10.1126/science.254.5029.277

[b3] Jullien D. . IL-15, an immunomodulator of T cell responses in intracellular infection. J. Immunol. Baltim. Md 1950 158, 800–806 (1997). 8992997

[b4] Michalany J. & Michalany N. S. A new morphological concept and classification of granulomatous inflammation: the polar granulomas. Ann. Pathol. 4, 85–95 (1984). 6375689

[b5] Virchow R. Die krankhaften Geschwülste. August Hirschwald 208 (1863).

[b6] Montoya D. . Divergence of macrophage phagocytic and antimicrobial programs in leprosy. Cell Host Microbe 6, 343–353 (2009). 19837374 10.1016/j.chom.2009.09.002 PMC2764558

[b7] Chehl S., Ruby J., Job C. K. & Hastings R. C. The growth of Mycobacterium leprae in nude mice. Lepr. Rev. 54, 283–304 (1983). 6230496 10.5935/0305-7518.19830035

[b8] Sibley L. D. & Krahenbuhl J. L. Mycobacterium leprae-burdened macrophages are refractory to activation by gamma interferon. Infect. Immun. 55, 446–450 (1987). 3100449 10.1128/iai.55.2.446-450.1987 PMC260348

[b9] Sibley L. D. & Krahenbuhl J. L. Induction of unresponsiveness to gamma interferon in macrophages infected with Mycobacterium leprae. Infect. Immun. 56, 1912–1919 (1988). 2840398 10.1128/iai.56.8.1912-1919.1988 PMC259501

[b10] Sibley L. D. & Krahenbuhl J. L. Defective activation of granuloma macrophages from Mycobacterium leprae-infected nude mice. J. Leukoc. Biol. 43, 60–66 (1988). 2826628 10.1002/jlb.43.1.60

[b11] Annunziata M., Granata R. & Ghigo E. The IGF system. Acta Diabetol. 48, 1–9 (2011). 21042815 10.1007/s00592-010-0227-z

[b12] Gomes C. M. . Insulin-like growth factor (IGF)-I affects parasite growth and host cell migration in experimental cutaneous leishmaniasis. Int. J. Exp. Pathol. 81, 249–255 (2000). 10971746 10.1046/j.1365-2613.2000.00157.x PMC2517735

[b13] Vendrame C. M. V. . Effect of insulin-like growth factor-I on Leishmania amazonensis promastigote arginase activation and reciprocal inhibition of NOS2 pathway in macrophage *in vitro*. Scand. J. Immunol. 66, 287–296 (2007). 17635806 10.1111/j.1365-3083.2007.01950.x

[b14] Rodrigues L. S. . Mycobacterium leprae induces insulin-like growth factor and promotes survival of Schwann cells upon serum withdrawal. Cell. Microbiol. 12, 42–54 (2010). 19732058 10.1111/j.1462-5822.2009.01377.x

[b15] Martinez F. O. & Gordon S. The M1 and M2 paradigm of macrophage activation: time for reassessment. F1000prime Rep. 6, 13 (2014). 24669294 10.12703/P6-13 PMC3944738

[b16] Moura D. F. . CD163 favors Mycobacterium leprae survival and persistence by promoting anti-inflammatory pathways in lepromatous macrophages. Eur. J. Immunol. 42, 2925–2936 (2012). 22851198 10.1002/eji.201142198

[b17] Denis M. Interferon-gamma-treated murine macrophages inhibit growth of tubercle bacilli via the generation of reactive nitrogen intermediates. Cell. Immunol. 132, 150–157 (1991). 1905984 10.1016/0008-8749(91)90014-3

[b18] Krahenbuhl J. & Adams L. B. Exploitation of gene knockout mice models to study the pathogenesis of leprosy. Lepr. Rev. 71 Suppl, S170–175 (2000). 11201877 10.5935/0305-7518.20000090

[b19] MacMicking J., Xie Q. W. & Nathan C. Nitric oxide and macrophage function. Annu. Rev. Immunol. 15, 323–350 (1997). 9143691 10.1146/annurev.immunol.15.1.323

[b20] Molloy A., Meyn P. A., Smith K. D. & Kaplan G. Recognition and destruction of Bacillus Calmette-Guerin-infected human monocytes. J. Exp. Med. 177, 1691–1698 (1993). 7684432 10.1084/jem.177.6.1691 PMC2191057

[b21] Jordao L., Bleck C. K. E., Mayorga L., Griffiths G. & Anes E. On the killing of mycobacteria by macrophages. Cell. Microbiol. 10, 529–548 (2008). 17986264 10.1111/j.1462-5822.2007.01067.x

[b22] Sinsimer D. . Mycobacterium leprae actively modulates the cytokine response in naive human monocytes. Infect. Immun. 78, 293–300 (2010). 19841079 10.1128/IAI.00816-09 PMC2798203

[b23] Guo Z., Shao L., Du Q., Park K. S. & Geller D. A. Identification of a classic cytokine-induced enhancer upstream in the human iNOS promoter. FASEB J. 21, 535–542 (2007). 17158780 10.1096/fj.06-6739com

[b24] Rojas M., Olivier M. & García L. F. Activation of JAK2/STAT1-alpha-dependent signaling events during Mycobacterium tuberculosis-induced macrophage apoptosis. Cell. Immunol. 217, 58–66 (2002). 12426001 10.1016/s0008-8749(02)00515-4

[b25] Dominici S. . Involvement of Stat1 in the Phagocytosis of M. avium. Clin. Dev. Immunol. 2012 (2012). 10.1155/2012/652683 PMC3395244 22811740

[b26] Gutierrez M. G. . NF-kappa B activation controls phagolysosome fusion-mediated killing of mycobacteria by macrophages. J. Immunol. Baltim. Md 1950 181, 2651–2663 (2008). 10.4049/jimmunol.181.4.2651 18684956

[b27] Pereira R. M. S. . Mycobacterium leprae induces NF-kappaB-dependent transcription repression in human Schwann cells. Biochem. Biophys. Res. Commun. 335, 20–26 (2005). 16055086 10.1016/j.bbrc.2005.07.061

[b28] Tanigawa K. . Expression of adipose differentiation-related protein (ADRP) and perilipin in macrophages infected with Mycobacterium leprae. FEMS Microbiol. Lett. 289, 72–79 (2008). 19054096 10.1111/j.1574-6968.2008.01369.x

[b29] Sato K., Akaki T. & Tomioka H. Differential potentiation of anti-mycobacterial activity and reactive nitrogen intermediate-producing ability of murine peritoneal macrophages activated by interferon-gamma (IFN-gamma) and tumour necrosis factor-alpha (TNF-alpha). Clin. Exp. Immunol. 112, 63–68 (1998). 9566791 10.1046/j.1365-2249.1998.00554.x PMC1904942

[b30] Herbst S., Schaible U. E. & Schneider B. E. Interferon gamma activated macrophages kill mycobacteria by nitric oxide induced apoptosis. PloS One 6, e19105 (2011). 21559306 10.1371/journal.pone.0019105 PMC3085516

[b31] Barrett J. P., Minogue A. M., Falvey A. & Lynch M. A. Involvement of IGF-1 and Akt in M1/M2 activation state in bone marrow-derived macrophages. Exp. Cell Res. 335, 258–268 (2015). 26022664 10.1016/j.yexcr.2015.05.015

[b32] Arkins S., Rebeiz N., Brunke-Reese D. L., Biragyn A. & Kelley K. W. Interferon-gamma inhibits macrophage insulin-like growth factor-I synthesis at the transcriptional level. Mol. Endocrinol. Baltim. Md 9, 350–360 (1995). 10.1210/mend.9.3.7776981 7776981

[b33] Yoshimura A., Suzuki M., Sakaguchi R., Hanada T. & Yasukawa H. SOCS, Inflammation, and Autoimmunity. Front. Immunol. 3 (2012). 10.3389/fimmu.2012.00020 PMC3342034 22566904

[b34] Ebong S., Yu C.-R., Carper D. A., Chepelinsky A. B. & Egwuagu C. E. Activation of STAT signaling pathways and induction of suppressors of cytokine signaling (SOCS) proteins in mammalian lens by growth factors. Invest. Ophthalmol. Vis. Sci. 45, 872–878 (2004). 14985304 10.1167/iovs.03-0311

[b35] Shen X., Hong F., Nguyen V. A. & Gao B. IL-10 attenuates IFN-alpha-activated STAT1 in the liver: involvement of SOCS2 and SOCS3. FEBS Lett. 480, 132–136 (2000). 11034314 10.1016/s0014-5793(00)01905-0

[b36] Niemand C. . Activation of STAT3 by IL-6 and IL-10 in primary human macrophages is differentially modulated by suppressor of cytokine signaling 3. J. Immunol. Baltim. Md 1950 170, 3263–3272 (2003). 10.4049/jimmunol.170.6.3263 12626585

[b37] Liu P. T., Stenger S., Tang D. H. & Modlin R. L. Cutting edge: vitamin D-mediated human antimicrobial activity against Mycobacterium tuberculosis is dependent on the induction of cathelicidin. J. Immunol. Baltim. Md 1950 179, 2060–2063 (2007). 10.4049/jimmunol.179.4.2060 17675463

[b38] Babon J. J. . Suppression of cytokine signaling by SOCS3: characterization of the mode of inhibition and the basis of its specificity. Immunity 36, 239–250 (2012). 22342841 10.1016/j.immuni.2011.12.015 PMC3299805

[b39] Qin H. . SOCS3 deficiency promotes M1 macrophage polarization and inflammation. J. Immunol. Baltim. Md 1950 189, 3439–3448 (2012). 10.4049/jimmunol.1201168 PMC4184888 22925925

[b40] Masood K. I. . Expression of M. tuberculosis-induced suppressor of cytokine signaling (SOCS) 1, SOCS3, FoxP3 and secretion of IL-6 associates with differing clinical severity of tuberculosis. BMC Infect. Dis. 13, 13 (2013). 23320781 10.1186/1471-2334-13-13 PMC3562147

[b41] Mattos K. A. . Lipid droplet formation in leprosy: Toll-like receptor-regulated organelles involved in eicosanoid formation and Mycobacterium leprae pathogenesis. J. Leukoc. Biol. 87, 371–384 (2010). 19952355 10.1189/jlb.0609433

[b42] Ge R.-T. . Insulin-like growth factor-1 endues monocytes with immune suppressive ability to inhibit inflammation in the intestine. Sci. Rep. 5, 7735 (2015). 25588622 10.1038/srep07735 PMC4295102

[b43] MacKenzie K. F. . PGE(2) induces macrophage IL-10 production and a regulatory-like phenotype via a protein kinase A-SIK-CRTC3 pathway. J. Immunol. Baltim. Md 1950 190, 565–577 (2013). 10.4049/jimmunol.1202462 PMC3620524 23241891

[b44] Wynes M. W. & Riches D. W. H. Induction of macrophage insulin-like growth factor-I expression by the Th2 cytokines IL-4 and IL-13. J. Immunol. Baltim. Md 1950 171, 3550–3559 (2003). 10.4049/jimmunol.171.7.3550 14500651

[b45] Rodrigues L. S. . Circulating levels of insulin-like growth factor-I (IGF-I) correlate with disease status in leprosy. BMC Infect. Dis. 11, 339 (2011). 22166091 10.1186/1471-2334-11-339 PMC3266221

[b46] Clemmons D. R. Modifying IGF1 activity: an approach to treat endocrine disorders, atherosclerosis and cancer. Nat. Rev. Drug Discov. 6, 821–833 (2007). 17906644 10.1038/nrd2359

[b47] Waters M. F. & Rees R. J. Changes in the morphology of Mycobacterium leprae in patients under treatment. Int. J. Lepr. 30, 266–277 (1962). 13999150

[b48] Cardoso C. C., Pereira A. C., de Sales Marques C. & Moraes M. O. Leprosy susceptibility: genetic variations regulate innate and adaptive immunity, and disease outcome. Future Microbiol. 6, 533–549 (2011). 21585261 10.2217/fmb.11.39

[b49] Osorio E. Y. . Growth factor and Th2 cytokine signaling pathways converge at STAT6 to promote arginase expression in progressive experimental visceral leishmaniasis. PLoS Pathog. 10, e1004165 (2014). 24967908 10.1371/journal.ppat.1004165 PMC4072777

[b50] Dalton J. E. . Inhibition of receptor tyrosine kinases restores immunocompetence and improves immune-dependent chemotherapy against experimental leishmaniasis in mice. J. Clin. Invest. 120, 1204–1216 (2010). 20234089 10.1172/JCI41281 PMC2846033

[b51] Ridley D. S. & Jopling W. H. Classification of leprosy according to immunity. A five-group system. Int. J. Lepr. Mycobact. Dis. Off. Organ Int. Lepr. Assoc. 34, 255–273 (1966). 5950347

[b52] Shepard C. C. & McRae D. H. A method for counting acid-fast bacteria. Int. J. Lepr. Mycobact. Dis. Off. Organ Int. Lepr. Assoc. 36, 78–82 (1968). 4869698

[b53] Moura D. F. . Long-term culture of multibacillary leprosy macrophages isolated from skin lesions: a new model to study Mycobacterium leprae-human cell interaction. Br. J. Dermatol. 157, 273–283 (2007). 17553031 10.1111/j.1365-2133.2007.07992.x

[b54] Wade H. W. Zenker vs formalin fixation for the histopathology of leprosy tissues and other desirable features of technique. Int. J. Lepr. 30, 477–488 (1962). 13998168

[b55] Vivarini A. de C. . Human cutaneous leishmaniasis: interferon-dependent expression of double-stranded RNA-dependent protein kinase (PKR) via TLR2. FASEB J. Off. Publ. Fed. Am. Soc. Exp. Biol. 25, 4162–4173 (2011). 10.1096/fj.11-185165 21846836

[b56] McNab F. W. . Type I IFN induces IL-10 production in an IL-27-independent manner and blocks responsiveness to IFN-γ for production of IL-12 and bacterial killing in Mycobacterium tuberculosis-infected macrophages. J. Immunol. Baltim. Md 1950 193, 3600–3612 (2014). 10.4049/jimmunol.1401088 PMC4170673 25187652

[b57] Martinez A. N. . Molecular determination of Mycobacterium leprae viability by use of real-time PCR. J. Clin. Microbiol. 47, 2124–2130 (2009). 19439537 10.1128/JCM.00512-09 PMC2708532

