# Supplementary material for: Mycobacterium leprae-induced Insulin-like Growth Factor I attenuates antimicrobial mechanisms, promoting bacterial survival in macrophages
Source: Sci Rep. 2016 Jun 10;6:27632. doi: 10.1038/srep27632 (PMC4901318; doi:10.1038/srep27632)
Supplement: Supplementary Information [file srep27632-s1.pdf]

Supplementary Information

Mycobacterium leprae-induced Insulin-like Growth Factor I attenuates antimicrobial mechanisms, promoting bacterial survival in macrophages

Leonardo Ribeiro Batista Silva[1¶], Luciana Silva Rodrigues[1¶*], Aislan de Carvalho Vivarini[2], Fabrício da Mota Ramalho Costa[1], Katherine Antunes de Mattos[1&], Maria Renata Sales Nogueira Costa[3], Patricia Sammarco Rosa[3], Thiago Gomes Toledo Pinto[4], André Alves Dias[1], Danielle Fonseca Moura[4], Euzenir Nunes Sarno[4], Ulisses Gazos Lopes[2], Maria Cristina Vidal Pessolani[1▲].

[1]Laboratory of Cellular Microbiology, Oswaldo Cruz Institute, Rio de Janeiro, 21040-900, RJ, Brazil;

[2]Laboratory of Molecular Parasitology, Carlos Chagas Filho Institute, Federal University of Rio de Janeiro (UFRJ), Rio de Janeiro, 21940-590, RJ, Brazil;

[3]Lauro de Souza Lima Institute, Bauru, 17034-971, SP, Brazil;

[4]Leprosy Laboratory, Oswaldo Cruz Institute, Rio de Janeiro, 21040-900, RJ, Brazil.

Present address: [*]Laboratory of Immunopathology, Rio de Janeiro State University (UERJ), Rio de Janeiro, 20550-170, RJ, Brazil; [&]Laboratory of Quality Control, Immunobiological Technology Institute, Bio-Manguinhos, Oswaldo Cruz Foundation, Rio de Janeiro, 21040-900, RJ, Brazil.

[▲] Correspondence should be addressed to: Dr. Pessolani, Laboratory of Cellular Microbiology, Oswaldo Cruz Institute, Oswaldo Cruz Foundation (FIOCRUZ), Rio de Janeiro, RJ, Brazil 21045-900; cpessola@ioc.fiocruz.br; Tel. (+55) 21 2562-1595.

¶ These authors contributed equally to this work.

Supplementary Figure 1

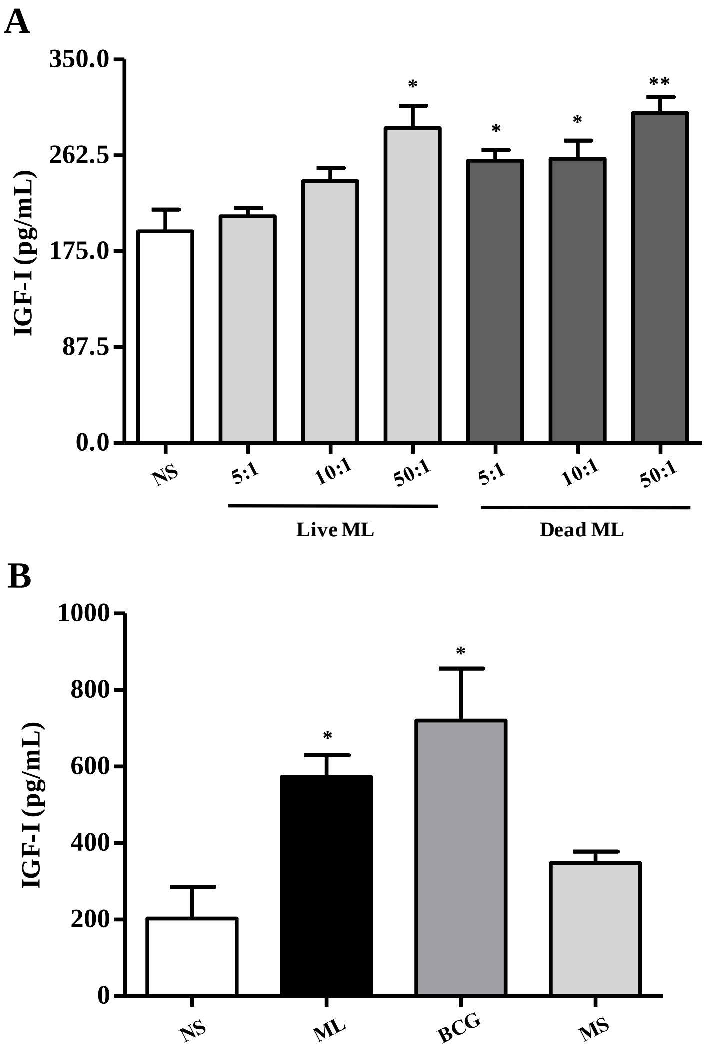

Supplementary Figure 1 │ IGF-I is produced by *in vitro* ML and BCG-stimulated macrophages, but not by *M. smegmatis* (MS). (A) RAW 264.7 macrophages were exposed to live or dead (irradiated) ML at different bacteria. ratios: cell ratio (5, 10 and 50) for 48h. IGF-I protein levels were measured in culture supernatants by specific sandwich ELISA. Data are shown as mean ± SE from six independent experiments performed in duplicate. (B) RAW 264.7

macrophages were exposed to dead ML, BCG, or MS at bacteria: cell ratio (50) for 48h. IGF-I protein levels were measured in culture supernatants by specific sandwich ELISA. Data are shown as mean ± SE from six independent experiments performed in duplicate. An ANOVA test followed by Bonferroni as a post test were performed and used for statistical analysis. *$p < 0.05$, **$p < 0.005$.

Supplementary Figure 2

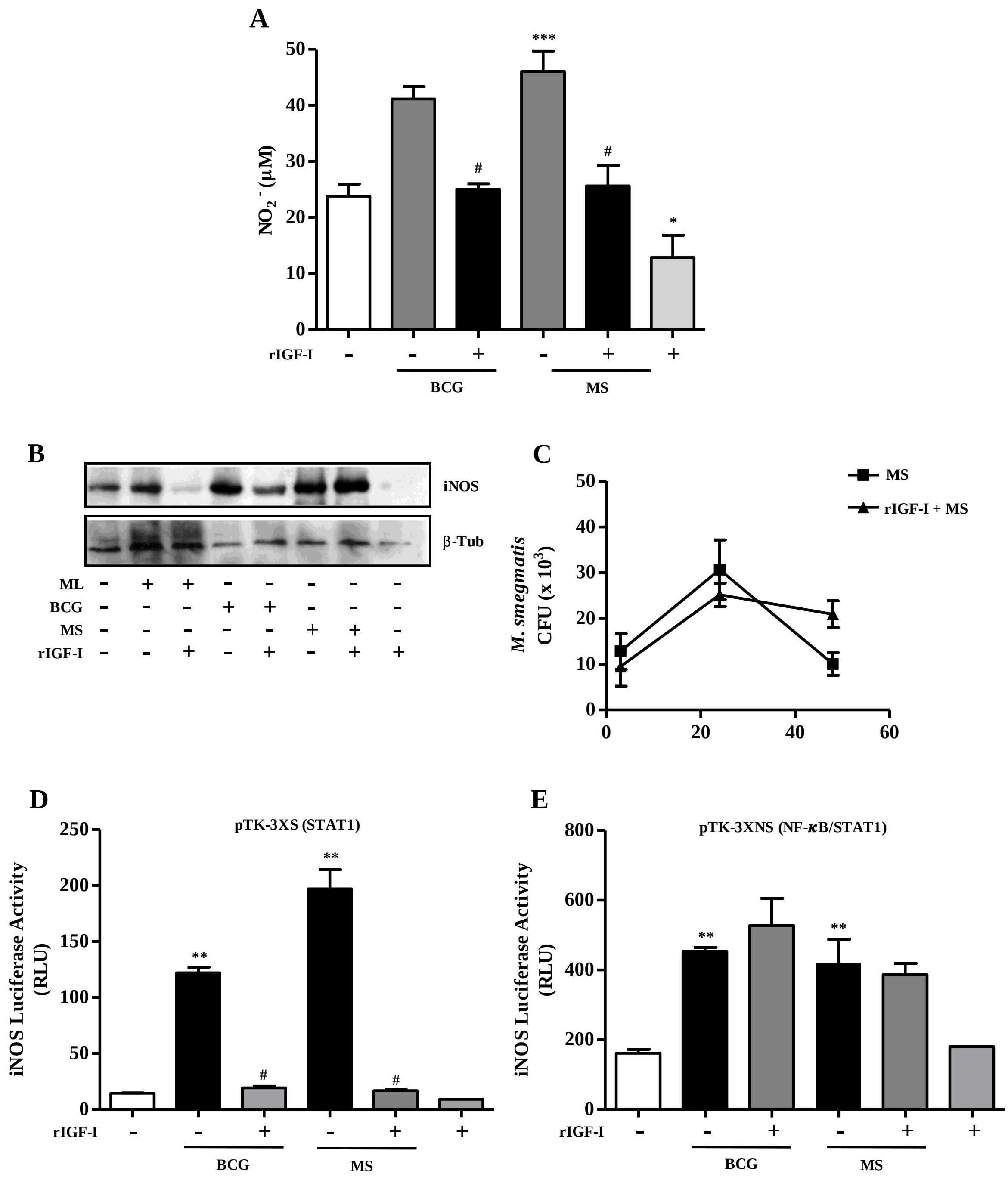

Supplementary Figure 2 │The macrophage microbicidal response to nonpathogenic mycobacteria is downregulated by IGF-I. (A) RAW 264.7 cultures were pre-treated with rIGF-I (50ng/ml) for 30 min and then exposed to irradiated BCG or MS at a bacteria: cell ratio of 50. After 48h stimulation, nitrite was measured in the supernatants by the Griess reagent. Each value represents the mean ± SE of three independent experiments performed in duplicate. (B) Total lysates from cultures were subjected to Western blot using the specific antibodies against iNOS and β-tubulin (β-Tub). The Figure shows a representative Western blot from three independent experiments. (D and E) RAW 264.7 cells transiently transfected with iNOS-luciferase reporter constructs pTK-3XS (D) or pTK-3XNS (E) were pre-treated with rIGF-I and then incubated with each mycobacterium for 24h, as described above. The cells were harvested and the luciferase activity determined by using the luciferase reporter assay system. Data are shown as mean ± SD of a representative experiment from three different experiments performed in triplicate. (C) Intracellular growth/survival of MS in macrophages pre-treated with rIGF-I was analyzed by the colony forming unit (CFU). Data are expressed as mean ± SD of the fold increase from three independent experiments performed in triplicate. An ANOVA test followed by a Bonferronipost test were performed and used for statistical analysis. * Statistically significant comparisons between unstimulated cultures and those incubated with mycobacterium. $^{\#}$Statistically significant comparisons between culture mycobacterium with mycobacteria in the presence or absence of rIGF-I.*$p < 0.05$, ***$p < 0.0001$, RLU, relative luminescence units.

Supplementary Figure 3

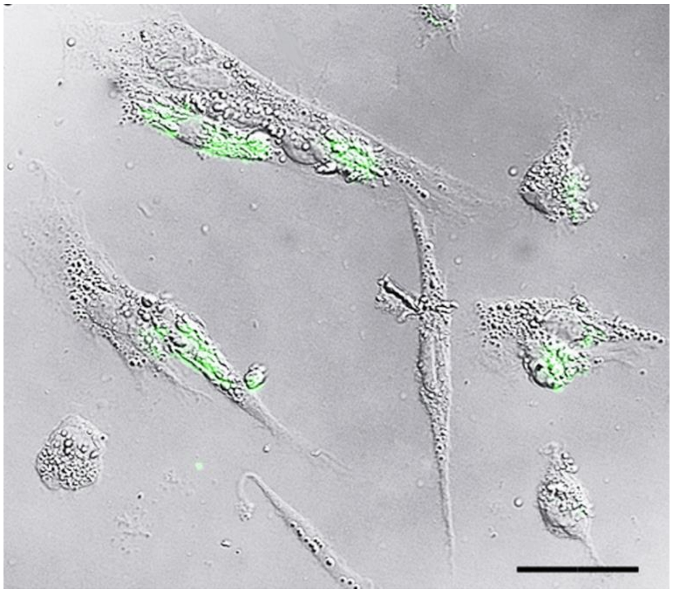

Supplementary Figure 3 │*In vivo* ML-infected macrophages isolated from skin lesions of LL patients. Fluorescence image of Virchow cells isolated from anLL skin lesion visualized by DIC and obtained by a Zeiss Colibri fluorescence microscope. The results shown are representative of a single experiment with three replicates/cultures. Scale bar = 100µm.
